# Clinical Characteristics and Outcome of Patients with Neuroblastoma Presenting Genomic Amplification of Loci Other than *MYCN*

**DOI:** 10.1371/journal.pone.0101990

**Published:** 2014-07-11

**Authors:** Anne Guimier, Sandrine Ferrand, Gaëlle Pierron, Jérôme Couturier, Isabelle Janoueix-Lerosey, Valérie Combaret, Véronique Mosseri, Estelle Thebaud, Marion Gambart, Dominique Plantaz, Aurélien Marabelle, Carole Coze, Xavier Rialland, Sylvie Fasola, Eve Lapouble, Paul Fréneaux, Michel Peuchmaur, Jean Michon, Olivier Delattre, Gudrun Schleiermacher

**Affiliations:** 1 Institut Curie, Département de Pédiatrie, Paris, France; 2 Institut Curie, Unité de Génétique Somatique, Paris, France; 3 INSERM U830, Laboratoire de Génétique et Biologie des Cancers, Institut Curie, Paris, France; 4 Centre Léon Bérard, Laboratoire de recherche translationnelle, Lyon, France; 5 Institut Curie, Service de biostatistiques, Paris, France; 6 CHU Nantes, Service d'Hémato-Oncologie Pédiatrique, Nantes, France; 7 CHU Toulouse, Service d'Hémato-Oncologie Pédiatrique, Toulouse, France; 8 CHU Grenoble, Service d'Hémato-Oncologie Pédiatrique, Grenoble, France; 9 Institut d'Hématologie et d'Oncologie Pédiatrique, Centre de Lutte contre le Cancer Léon Bérard, Lyon, France; 10 Aix-Marseille Univ et APHM, Hôpital d'Enfants de La Timone, Service d'Hématologie-Oncologie Pédiatrique, Marseille, France; 11 CHU Angers, Service d'Hémato-Oncologie Pédiatrique, Angers, France; 12 Hôpital Trousseau, Service d'Hémato-Oncologie Pédiatrique, Paris, France; 13 Institut Curie, Laboratoire d'anatomie pathologique, Paris, France; 14 APHP, hôpital Universitaire Robert Debré, Service de Pathologie, Paris, France, et Université Diderot Paris 7, Sorbonne Paris Cité, Paris, France; The Institute of Cancer Research, London, United Kingdom

## Abstract

**Background:**

Somatically acquired genomic alterations with *MYCN* amplification (MNA) are key features of neuroblastoma (NB), the most common extra-cranial malignant tumour of childhood. Little is known about the frequency, clinical characteristics and outcome of NBs harbouring genomic amplification(s) distinct from *MYCN*.

**Methods:**

Genomic profiles of 1100 NBs from French centres studied by array-CGH were re-examined specifically to identify regional amplifications. Patients were included if amplifications distinct from the *MYCN* locus were seen. A subset of NBs treated at Institut Curie and harbouring MNA as determined by array-CGH without other amplification was also studied. Clinical and histology data were retrospectively collected.

**Results:**

In total, 56 patients were included and categorised into 3 groups. Group 1 (n = 8) presented regional amplification(s) without MNA. Locus 12q13-14 was a recurrent amplified region (4/8 cases). This group was heterogeneous in terms of INSS stages, primary localisations and histology, with atypical clinical features. Group 2 (n = 26) had MNA as well as other regional amplifications. These patients shared clinical features of those of a group of NBs *MYCN* amplified (Group 3, n = 22). Overall survival for group 1 was better than that of groups 2 and 3 (5 year OS: 87.5%±11% vs 34.9%±7%, *log-rank p<0.05*).

**Conclusion:**

NBs harbouring regional amplification(s) without MNA are rare and seem to show atypical features in clinical presentation and genomic profile. Further high resolution genetic explorations are justified in this heterogeneous group, especially when considering these alterations as predictive markers for targeted therapy.

## Introduction

Neuroblastoma (NB) is the most common extra-cranial malignant tumour of childhood, [1] and is characterised by its wide heterogeneity in clinical presentation and evolution [1]–[3]. Recent advances in genetic analysis of this heterogeneous tumour, using a wide panel of techniques including array Comparative Genomic Hybridization (aCGH), have revealed different recurrent genomic aberrations, most of which consist of copy number alterations. Indeed, it is now well established that the overall genomic pattern is an important prognostic marker which might be taken into account for treatment stratification [4]–[13]. Numerical chromosome alterations (whole chromosome gains or losses) are observed in NBs with good prognosis when exclusive. Typical segmental copy number alterations (deletions of chromosome arms 1p, 3p, 4p, 11q and gains of chromosome arms 1q, 2p, 17q) are associated with poor outcome [7].

Amplification of the proto-oncogene *MYCN* (MNA), found in 25 to 30% of NBs, is the most important genomic feature, in terms of prognosis and impact on treatment decisions [2], [3]. Other genomic aberrations defined by regional amplifications targeting various sites, non syntenic with the *MYCN* locus, have been previously described [5], [6], [14]–[19]. These amplicons seem to have a low recurrence and most often occur concomitantly with MNA [16]. In a previous study, the precise genetic mapping of such amplicons has been described, and a poor survival for patients with NBs harbouring loci co-amplified or not with *MYCN* has been suggested [16].

Nevertheless, to date, clinical features of NB harbouring amplicons different from *MYCN*, and particularly without concurrent MNA, have not yet been reported in detail. The role of these amplicons and their possible contribution to the oncogenic process are unclear and there is a need to better characterise clinically these tumours. The aim of this study is to describe occurrence, detailed clinical characteristics, histology and outcome of NBs harbouring amplicons at loci distinct from *MYCN*, without and with MNA.

## Patients and Methods

### Ethics statement

This study was authorized and approved by the ethics committee “Comité de Protection des Personnes Sud-Est IV”, reference L07-95 and L12–171 and the ethical committee Ile de France, reference 0811728. Written informed consent was obtained from parents according to national law and the ethics committees listed above approved this consent procedure.

### Patients and samples

Between 1996 and 2011, tumour samples from a total of 1100 patients were sent to the laboratory from French centres for genetic analysis and were studied by aCGH.

The 1100 aCGH profiles were re-examined specifically for high level amplification, regardless of their overall genomic pattern, using the VAMP graphical interface and visual inspection [20]. aCGH profiles were taken into account if tumour cell content was known to be >50%, or in the absence of known tumour cell content, if the copy number profile showed a clear dynamic profile. All patients for whom amplifications distinct from the *MYCN* locus were observed were then selected. As a control group, patients treated at Institut Curie from 1999 to 2011, for whom aCGH was performed on the tumour and revealed a MNA without any other amplification were also studied. The aCGH platform and analysis steps were identical for all patients.

A total of 56 patients were included in this study. For all cases *MYCN* status was confirmed by Fluorescent in situ hybridization (FISH) [21] and no discordance between aCGH and FISH has been observed. Clinical data (sex, date of birth, date at diagnosis, INSS tumour stage, localisation, MIBG uptake, sequence of treatment, relapse or progression, date and status of last follow up), biological data (urinary catecholamine at diagnosis) and histology (tumours were classified according to INPC, with focus on the differentiation of the tumour cells) were retrospectively collected. Patients were treated in French centres from the Société Française des Cancers de l'Enfant (SFCE) according to national or international treatment protocols.

### Comparative Genomic Hybridization and definition of genomic amplification

Tumour samples sent to the laboratory for somatic pangenomic analysis were studied by aCGH as previously described [16]. The resolution was determined by the genomic spacing of the array elements. Two types of arrays have been used: until 2009 an in-house designed array containing between 2855 and 3799 BAC-PAC clones covering the whole genome with a median probe spacing of 1 Mb [16], then a commercial array (NimbleGen) was used with an average resolution of 40 kb (72 000 probes).

Amplification was defined by at least two BAC clones (for the in-house array) or at least 3 adjacent oligonucleotide probes (for the NimbleGen array taking into account its higher resolution) with a fluorescent tumour/normal ratio ≥3 corresponding to a log2 ratio ≥1, 5 [16]. Boundaries of an amplicon were described according to the genomic position of the markers located outside the amplified region (coordinates of the non amplified markers closest to the observed amplicon, according to UCSC genome draft, hg19 (http://genome.ucsc.edu/)). On chromosome band 2p24, amplicons harbouring *MYCN* with or without directly adjacent co-amplified genes were considered as MNA. In case of amplifications of loci distant from *MYCN* but still within the cytogenetic 2p24 band, we defined arbitrarily an amplicon distinct from *MYCN*, when the amplicon was separated from *MYCN* locus by at least five BAC/PAC clones with a normal fluorescence tumour/normal ratio on the in-house array, or the corresponding number of probes on the NimbleGen array.

Analysis of somatic genetic alterations with definition of losses, gains and high level amplifications was performed as previously described [16]. Precise genetic analysis of 31 of the NBs in this study has been reported previously [16] but the precise clinical data had not been analysed for these patients.

### Statistical analysis

Contingency tables were analysed by the Fisher exact test. Mean values were compared by the non parametric Kruskal Wallis Test. Median follow up was calculated according to the inversed Kaplan-Meier method. Progression free survival (PFS) was defined as the time between diagnosis and the first event: relapse, progression, and death from any cause or last follow-up. Overall survival (OS) was defined as the time between diagnosis and death of any cause or last follow-up. Survival curves were analyzed according to the Kaplan-Meier method and compared using the log-rank test with a P-value of less than 0.05 considered to be significant.

## Results

### Genomic amplifications

**Figure 1:**
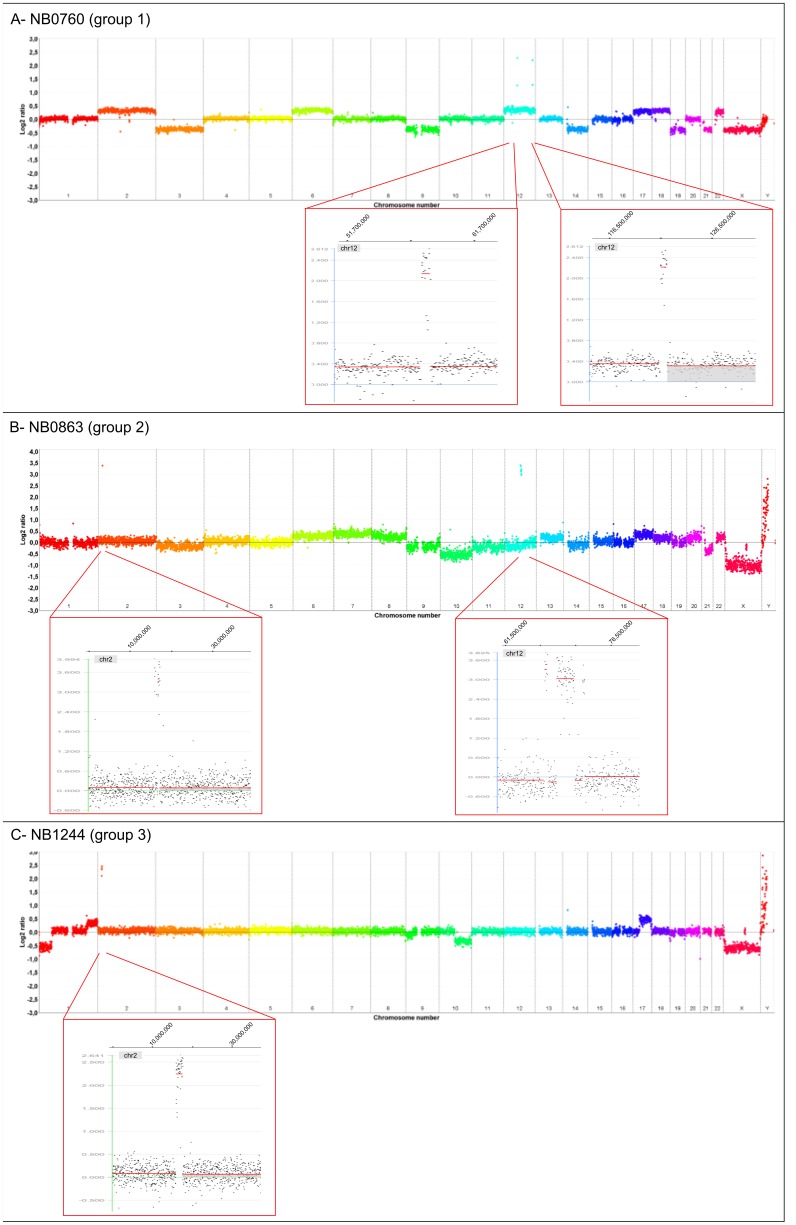
Examples of different genomic profiles with genomic amplifications obtained in neuroblastoma by array comparative genomic hybridization for each group at diagnosis. For each panel, the genome wide aCGH profile is shown with zoom on the amplified regions. Genomic profiles were obtained using the NimbleGen R platform, and images were generated using the SignalMap R software. Log2 ratio  = 0 corresponds to a balanced tumour/normal DNA ratio. Amplification is indicated by plots with a log2 ratio ≥1, 5 (corresponding to a tumour/normal DNA ratio ≥3). (A) Example of group 1 profile (NB0760): NB without *MYCN* amplification but harbouring amplifications at loci 12q13-14 and 12q24. (B) Example of group 2 profile (NB0863): NB with *MYCN* amplification (2p24) and harbouring amplification at locus 12q14. (C) Example of group 3 profile (NB1244): NB with *MYCN* amplification and no other amplicon.

Among 1100 aCGH profiles, we found a total of 12 tumours showing amplification of one or several loci distinct from *MYCN* without any evidence of MNA (1%). We did not get access to clinical information for two of them. For another two, the histological report was non conclusive and a definitive diagnosis of NB could not be retained. Indeed, diagnosis of malignant pheochromocytoma was suggested. These four patients were not included. Therefore eight confirmed NBs presenting regional amplification(s), without MNA, were included as group 1 (n = 8). A further 26 patients were found with NB exhibiting MNA associated with one or several amplification(s) at other loci (group 2, n = 26). Finally, 22 patients treated for NB in our institution between 1999 and 2011, for whom aCGH have been performed on tumour, were identified with amplification only located at the *MYCN* locus and were considered as a control group (group 3, n = 22). Examples of genomic profile for each of the 3 groups are shown in **Figure 1** with zoom on the amplified regions. Altogether, the most frequent amplified regions distinct from MNA were located at chromosome band 19p12, 2p25, 2p23, 21q21, 22q11 and 12q13–14, with amplification in other chromosome regions being much rarer. Genomic findings for groups 1 and 2 cases are summarised in **Table 1** **and** **Table 2** respectively. Precise amplification boundaries are available in Table S1 and in a.BED file (File S1) enabling to export all genes possibly included in the amplicons, according to UCSC genome draft, hg19 (http://genome.ucsc.edu/).

**Table 1:** Clinical characteristics, genomic findings and histology data for group 1.

Patient	Clinical characteristics										Genomic findings		Histology
	Sex	Age at diagnosis (months)	INSS stage	Localisation	Metastasis at diagnosis	MIBG uptake	Urinary catechol	Initial treatment	Relapse (months)	Outcome (FU months)	Amplicons (cytogenetic band)	Segmental alterations	Grade of differentiation
NB0384	M	23	4	Abd	B, M	Y	H	IM	No	CR(121)	11q13	−1p, −3p, −11q, +17q	P. diff NB
NB0040	M	54	4	Adr	B, M, DLN	Y	H	IM	Ms(25)	DOD(75,5)	11q13, 17q25	−11q, −16p, −16q, +17q	P. diff NB
NB0791	M	18	3	Cervical	LLN	No	Nl	CT-S	No	CR(44)	16q22	numerical	Undiff NB
NB0760	F	17	4	Abd	DLN	Y	H	IM	No	CR(44)	12q13_14, 12q24	numerical	P. diff NB
NB0830	F	22	2b	Abd median	none	No	Nl	S	No	CR(27)	12q13_14	−22q	Diff NB
NB0037	M	55	3	Abd lumbar	LLN	Y	H	S-CT	L(7)	CR(129)	12q13_15, 21q22	+1p, +13q, +17q	P. diff NB
NB0072	F	51	4	Adr	B, M, DLN	Y	na	CT-S	No	Al(31)	7q21	−3p, +18 pq	Undiff NB
NB0039	M	67	4	Adr	Lung	No	Nl	IM	Ms+L(14,5)	DOD(19)	12q12, 12q13_15	+1p, +4q	P.diff NB

M, male; F, female; Adr, adrenal; Abd, abdominal; B, bone; M: bone marrow; DLN: distant lymph nodes; LLN: local lymph nodes; Ms, Metastatic relapse; L, Local relapse; MIBG, metaiodobenzylguanidine uptake at primary tumour site; Y, yes; H, high secretion of urinary catecholamines; Nl, normal secretion of urinary catecholamines; IM, intensive multimodality; S: surgery; CT, chemotherapy; DOD, dead of disease; Al, alive status unknown; CR, complete remission; NB, neuroblastoma; Undiff., undifferentiated; P.diff., poorly differentiated; Diff, differentiating; na, not available.

**Table 2:** Clinical characteristics, genomic findings and histology data for group 2.

Patient	Clinical characteristics								Genomic findings		Histology
	Sex	Age at dg (months)	INSS stage	Location	MIBG uptake	Urinary catechol	Relapse (months)	Outcome (FU months)	Amplicons (cytogenetic band)	Segmental alterations	
NB0187	M	25	3	Adr	Y	na	No	CR (107)	19p12, 21q21	−1p, −15q, +17q	NB NOS
NB1085	M	27	4	Adr	Y	H	No	CR (18)	1p31, 2p23 (*ALK*), 4q13	−1p, +17q	Undiff NB
NB0240	F	26	1	Adr	Y	H	L(23)	DOD (25)	1p36.3	−1p, +2p, +17q	P. diff NB
NB0038	F	25	4	Pelvic	Y	H	L(12)	DOD (15)	2p23 (*ALK*), 8q12, 17q23, 19p12, 21q21, 21q22, 22q11	−1p	Undiff NB
NB0284	M	43	3	Adr	Y	H	L(5,5)	DOD (6)	1p34.2, 1q32	+2p, −5q	NB NOS
NB0194	F	39	4	Abd +mediastinum	Y	H	Ms+L(7,5)	DOD (10)	2q35, 6p21	−1p, +12q, −10p, +17q	NB NOS
NB0186	M	9	4	Adr	Y	H	No	D tox (6)	22q11	−1p, +1q, −2p, +17q	P. diff NB
NB0196	M	24	4	Adr	Y	H	Ms+L(3,5)	DOD (4)	17q22, 17q25	+11q13 int, −17p, +17q	GGNB
NB0234	F	9	2b	Adr	Y	H	No	RC (156)	16q22_23	none	NB NOS
NB0185	F	15	3	Adr	Y	H	L(15,5)	DOD (19)	21q21, 22q11	−1p, +1q, +4q, −5q, −10p, −10q, +12q, +15q, +17q, +18q, −19q	Undiff NB
NB0232	M	17	4	Adr	Y	H	No	CR (103)	7q22,7q33_34, 7q36	−1p, +2q, −10q, +17q	P. diff NB
NB0260	M	19	4	Adr	Y	H	Ms+L(23)	DOD (25)	19p12	−1p, +3p, −5q, +7q, −16q, −17p, +17q, −19q	NB NOS
NB0236	M	21	4	Thoraco-abd-pelvic	Y	H	Ms(7)	DOD (9)	1p13, 6p23–24, 19p12, 21q21, 22q11	−1p, +17q	Undiff NB
NB0015	M	25	4	Adr	Y	H	No	CR (104)	19p12	−1p, +7pq, +17q	P. diff NB
NB0862	F	51	3	Adr	Y	H	No	CR (43)	5p15, 5q11	+17q	P. diff NB
NB1015	M	29	4	Adr	Y	na	Ms(8,5)	DOD (12)	2p25 (*ODC1*)	none	P. diff NB
NB0863	M	19	4	Abd	Y	H	Ms+L(14)	DOD (15)	12q14	numerical	P. diff NB
NB0230	M	44	4	Adr	Y	H	Ms(14)	DOD (19,5)	1p13, 2p23 (*ALK*), 2p25,19p12	−1p, +6p, −6q, +11q, +17q	P. diff NB
NB0256	M	16	4	Adr	Y	na	Ms(58)	DOD (65,4)	19p12, 21q21	−1p, +9q, −17p, +17q	P. diff NB
NB0173	M	6	4S	Adr	na	na	Ms+L(42)	DOD (49)	19q13.4	−1p, +17q	P. diff NB
NB0248	F	14	3	Adr	Y	Nl	No	CR (96)	17q23, 19p12	−1p, −7q, −21q, +17q	P. diff NB
NB1173	M	27	4	Adr	Y	na	Ms+L(11)	DOD (13)	12q13–14, 12q14_15	−1p, −12q, +17q	NB NOS
NB0013	F	13	4	Adr	Y	na	Ms(10)	DOD (10)	2p24_25 (ODC1)	−1p, −6q,+2p, +3q, +4q, +13q, +17q	NB NOS
NB1250	F	56	4	Adr	Y	H	Ms(6)	DOD(10)	2p25.2	−1p, −6q,+11q, +17q	P. diff NB
NB1257	F	149	4	Adr	Y	H	Ms+L(6,5)	DOD (17)	2p23 (ALK), 2q22	−1p, −5q, +6p, +7p, +7q, −10q, −12p, −14q, −15p, +17q, −18q	P. diff NB
NB0838	M	8	4	Adr	Y	H	Ms(4)	DOD (5,5)	2p25.1 (ODC1), 2p23 (ALK)	−1p	P. diff NB

M, male; F, female; dg, diagnosis; Adr, adrenal; Abd, abdominal;; MIBG, metaiodobenzylguanidine uptake at primary tumour site; Y, yes; H, high secretion of urinary catecholamines; Nl, normal secretion of urinary catecholamines; Ms, Metastatic relapse; L, Local relapse; DOD, dead of disease; D tox, dead of toxicity; CR, complete remission; NB, neuroblastoma; GGNB, ganglioneuroblastoma; Undiff., undifferentiated; P.diff., poorly differentiated; Diff, differentiating; NOS, not otherwise specified.

In group 1, the amplified region 12q13–14 was recurrent, observed in 4/8 cases (NB0760, NB0830, NB0037, NB0039). For two of them, the amplification was larger and comprises chromosome bands 12q13 to 12q15. Amplification at 11q13 was observed in two cases (NB0384, NB0040). Four NBs had a single amplification and the other four (NB0037, NB0039, NB0040, NB0760) had two distinct amplicons. These amplifications arise in an overall genomic pattern of segmental aberrations (**Table 1**). However for 4/8 cases, aCGH showed overall atypical genomic profiles for a NB, and for two others the genomic profile was numerical.

In group 2, more than 50% of cases had at least two regions co-amplified with MNA. Among these, amplification at chromosome band 19p12 was found in 8/26 cases, amplification of *ALK* at band 2p23 in 5/26 cases, and *ODC1* at band 2p25 in 3/26 cases (**Table 2** **and Table S1**). The majority of tumours in group 2 had a genomic profile typical of NB, with segmental chromosome alterations including losses of 1p, 11q and gain of 17q. One had only numerical chromosome alterations and two other cases neither numerical nor segmental chromosome alterations. In this group, aCGH profiles globally showed a higher number of copy number alterations (**Table 2**).

In group 3, aCGH profiles showed a single amplification at the *MYCN* locus associated with segmental chromosomal alterations typical of NB in most cases.

### Clinical characteristics

All patients from the 3 groups presented non familial and non syndromic NB. Detailed clinical characteristics and histology data for patients of groups 1 and 2 are summarised in **Tables 1** **and** **2** respectively.

For group 1, patients were of all INSS stages, the primary site occurred in different localisations, clinical features and histology were heterogeneous (**Table 1**). Interestingly, three patients (NB0791, NB0830, NB0039) in this group had normal urinary catecholamines and no uptake of MIBG at scintigraphy at diagnosis. For NB0039, lung metastasis was observed at diagnosis. Of note, NB0037 had an atypical presentation with a lumbar primary site with metastatic relapse located at the spermatic cord.

Patients in groups 2 (**Table 2**) and 3 (data not shown) shared clinical features with NB of advanced stage. All patients in group 2, except one (data not available), had positive MIBG uptake in their primary tumour. All except one (and data were not available for six patients) presented with high level urinary catecholamines. The primary tumour was localised mostly at the adrenal gland and histology was in general poorly differentiated or undifferentiated.

Concerning the general distribution of clinical parameters for the three groups (**Table 3**), no statistically significant difference of median age at diagnosis was found between the three groups, with an overall median age of 25 months. No difference was found between the three groups concerning distribution of INSS stage at diagnosis, with a high percentage of advanced stages observed in all three groups (Fisher exact test, *P* = 0.35). Interestingly, distribution of primary tumour localisation was different between group 1 and the two other groups (Fisher exact test, *P*<0.001). Indeed, localisation was mainly abdominal for the three groups, but an adrenal site was found for 37.5% of primary tumours in group 1 versus 85% in group 2 and 100% in group 3 (**Table 3**).

**Table 3:** Distribution of clinical parameters for the three groups.

Parameters		Group 1		Group 2		Group 3			
		n = 8	%	n = 26	%	n = 22	%	Total	*P*
Tumour INSS Stage	4	5	62,5	18	69	19	86	42	*NS*
	3	2	25	5	19	3	14	10	
	1, 2, 4S	1	12,5	3	12	0	0	4	
Median age at dg (months)[range]		37 [17–67]		24,5 [6–149]		29 [8–73]			*NS*
Age at dg	≤18 months	2	25	9	35	9	41	20	*NS*
	>18 months	6	75	17	65	13	59	36	
Tumour localisation	Adrenal	3	37,5	22	85	22	100	47	*<0.001*
	Other abdominal	4	50	3	11	0	0	7	
	Non abdominal	1	12,5	1	4	0	0	2	
Relapse		3	37,5	18	69	9	41	30	*NS*
	Metastatic +local	1		7		6		14	
	Metastatic only	1		7		3		11	
	Local only	1		4		0		5	

INSS, international neuroblastoma staging system; dg, diagnosis; *P*, Fisher's exact test p-value; NS, not significant.

### Survival analysis

**Figure 2:**
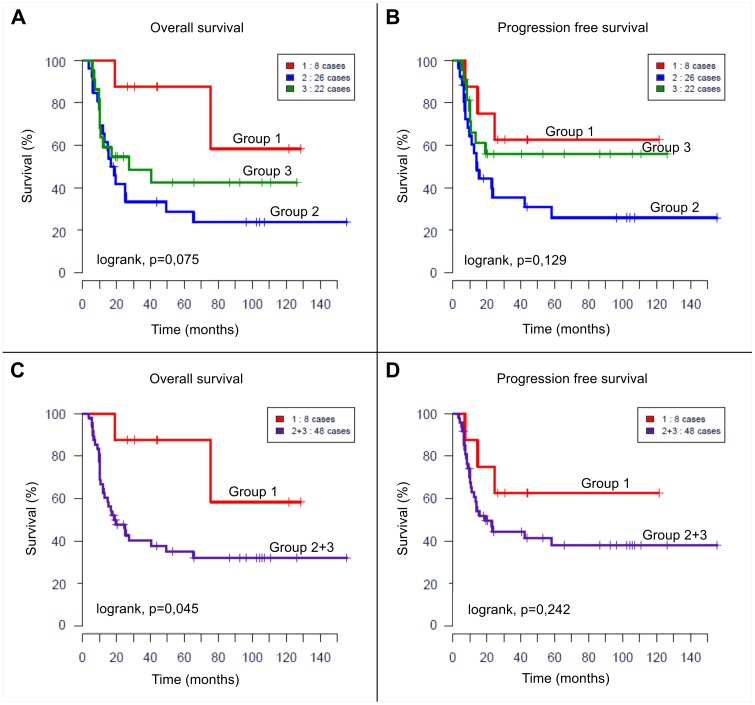
Kaplan Meier survival curves. (**A**, **B**) Overall survival and progression free survival curves of patients according to the 3 groups: group 1 (amplicon other than *MYCN*, not *MYCN* amplified, n = 8), group 2 (*MYCN* and other amplicons, n = 26), group 3 (only *MYCN* amplification, n = 22). Five-year OS rate was 87.5%±11.7%, 28.6%±9.2% and 42.4%±11.2% for groups 1, 2 and 3, respectively (*P* = 0.075). Five-year PFS rate was 62.5%±17%, 25.8%±9.2%, 55.9%±11% for groups 1, 2 and 3 respectively (*P* = 0.129). (**C**, **D**) Overall survival and progression free survival curves respectively for patients presenting NBs with amplification other than *MYCN*, without *MYCN* (group 1, n = 8) and patients with at least amplification of *MYCN* (group 2 and 3 pooled together, n = 48). Five-year OS rate was 87.5%±11.7% for group 1 and 34.9%±7% for group 2+3 (*P* = 0.045). Five-year PFS rate was 62.5%±17% for group 1 and 37.9%±7.7% for group 2+3 (*P* = 0.242).

Overall median follow up was 88 months (range from 3.7 to 155 months). No significant difference in outcome was found between the three groups (**Figure 2A and 2B**).

However when comparing patients of group 1 to all patients with NB harbouring *MYCN* amplification, i.e. groups 2 and 3 pooled together, we found a significantly better OS for group 1 (**Figure 2C, ***P* = 0, 045). There was no difference in PFS (**Figure 2D**).

Concerning groups 2 and 3, no statistically significant difference in outcome was found between patients with exclusively *MYCN* amplified NB and patients with MNA associated with other amplicons. Nevertheless a trend towards a poorer outcome in OS and particularly in PFS was observed for group 2 compared to group 3.

## Discussion

Somatic DNA amplification plays an important role in the development of many solid tumours possibly by providing a means of overexpression of oncogenes [22], [23]. Identification of high level amplifications has clinical impact due to the role of such genetic alterations as both prognostic and predictive molecular markers. *MYCN*, first identified in NB, was the first amplified proto-oncogene with significant clinical relevance, and its status is routinely used to stratify treatment [2], [3], [24], [25]. In other cancer types, such as medulloblastoma, amplification of MYC family genes is associated with clinical high risk disease and predicts an extremely poor prognosis [26]. Other specific gene amplifications, for instance in breast cancer such as *ERBB2* or *MDM2* are associated with high grade cancer and have strong prognostic significance [23], [27].

In NB, in previous studies, regional amplifications other than *MYCN* have been occasionally described with low recurrence and most often concomitantly with MNA [5], [6], [14], [17]–[19]. The objective of this study was now to report in detail the clinical features of patients with NB harbouring amplification(s) other than *MYCN*.

In this more extensive series, we confirm the rarity of regional amplifications occurring either without MNA or together with MNA (1% and 3% of all cases, respectively) as detected by aCGH. Given the resolution of the arrays used in this study, it cannot be excluded that amplified regions smaller than the interval between the probes of the arrays might have gone undetected by our techniques. However only few amplified regions distinct from *MYCN* have been observed in recent high resolution sequencing studies based on whole exome/genome sequencing [28]–[30], confirming that this is a rare phenomenon. Thus our lower resolution approaches give a good overview of the majority of amplicons in the genome. In a next step, it will be interesting to determine with accuracy genes implicated in the amplicons using next generation sequencing.

Interestingly, patients with NBs harbouring amplifications other than *MYCN*, without concomitant MNA, constitute a heterogeneous group of patients with NBs arising from non adrenal sites observed more frequently, as well as occurrence of atypical metastatic sites (lung, spermatic cord). Furthermore an increased frequency of absence of MIBG avidity and absence of urinary catecholamine secretion was noted, when normally positive in 90–95% of NB cases [1]. In addition to atypical clinical features, the overall genomic pattern of these NBs revealed atypical segmental patterns. Although histological analysis confirmed the diagnosis of NB, novel histology characterisation using PHOX2B immuno-staining might be useful in this context of atypical NB to help in the diagnosis of undifferentiated types [31]. Indeed PHOX2B immunolabelling has been shown to improve the diagnosis of undifferentiated NB among childhood small round blue-cell tumours with high specificity and sensitivity. Considering recent publication, it would be also interesting for this atypical group of NB without MNA to further study expression of MYC protein in the tumour as it has been suggested that MYC protein expression could be a new prognostic factor indicating more aggressive clinical behaviour than MNA [32].

On the other hand, clinical features of patients whose tumours harbour regional amplifications other than *MYCN* together with MNA are comparable to those with MNA only.

Although limited by the small number of patients, analysis suggests that OS of patients with amplification(s) other than *MYCN* without MNA might be better than that of patients with MNA, whereas those harbouring both *MYCN* and other amplifications might have an even worse prognosis. Indeed tumours harbouring regional amplicons in addition to MNA showed a higher genomic instability as documented by the observation of more segmental chromosomal alterations with a tendency towards a poorer outcome, as suggested previously [16]. Furthermore when comparing OS to a group of 170 NBs with segmental chromosomal alterations but without *MYCN* or other amplification (corresponding to genomic type B and D from a previous study [7]), the OS for group 1 was comparable to these NBs (type B and D) with 5 year OS of 87, 5%±11, 7 SE for group 1 vs 73%±3,9 SE and this result was significantly better than OS for patients in groups 2 and 3.

The genes targeted by regional amplifications in NB have been analysed in detail in previous study [5], [6], [9], [14], [16]–[19]. The most frequent amplifications concern *ALK* amplification at band 2p23, frequently co-amplified with *MYCN*, accounting for 4% of NBs studied in a meta-analysis [33]–[35], found in five cases in our study (group 2) and *ODC1* amplification at band 2p25 always found co-amplified with *MYCN* (20% of cases analysed in 2 studies) [9], [30], found in three cases in our study (group 2). Somatic amplification at 12q13–15 locus has also been described [6], [9], [12], [14]–[16], [19], [36]. This amplified region contains two potential target genes: *CDK4* (12q13_14) involved in cell cycle progression and *MDM2* (12q15), a target gene of the transcription factor tumour protein p53 and the encoded protein can target p53 for proteasomal degradation. In our study the amplicons at 12q13–14 and 12q13–15 were the most commonly amplified region in the absence of MNA with the *CDK4* gene amplified constantly but *MDM2* found amplified only in half of the cases. Amplifications at 12q13–14 and 12q13–15 have been reported in many other solid tumours such as malignant glioma, bladder cancer and sarcomas most often resulting in overexpression of genes in this region, with the implication of a worse prognosis in amplified cases [37]–[41]. *CDK6* gene at 7q21 was also amplified in the absence of MNA in one case (NB0072) and *CCND1* gene at 11q13 in two cases (NB0040 and NB0384). These observations are noteworthy considering that *CCND1*, *CDK4* and *CDK6* are G1 phase-regulating genes, part of the Cyclin D/CDK4/CDK6/RB pathway found hyperactive in NB, and considering the efficacy of new small molecule inhibitor targeting CDK4/CDK6 leading to G1 arrest and cellular senescence [42]. In group 1, without MNA, seven cases among eight presented amplification containing one of these genes.

Characterisation of amplicons using aCGH data combined with gene expression profiling analysis has shown that up to 25% of the genes targeted by genomic amplification are overexpressed in tumour cells, with a potential oncogenetic role, an observation of clinical importance when considering targeted therapies [16], [42].

Taken together, NB harbouring distinct amplification other than *MYCN* might have atypical clinical and genetic features and will warrant further studies including high resolution genomic analysis and expression data to more precisely characterise their genetic features and impact in oncogenic process. As more targeted therapies become available, such as molecules inhibiting specifically CDK4-CDK6 [42] or ALK for instance, it will be crucial to obtain precise information about genomic amplification status at diagnosis. As higher resolution genomic profiles are obtained, smaller genomically amplified regions might be detected more frequently. The prognostic impact of the amplified regions will have to be studied prospectively and their use as predictive markers for targeted therapy will be of importance.

## Supporting Information

Table S1 **Boundaries for amplifications found in group 1 and in group 2.** Boundaries of one amplified region are given according to the genomic position of the markers (BAC or oligonucleotide probe) located outside the amplified region (coordinates of the non amplified markers closest to the observed amplicon) and according to UCSC genome draft, hg19 (http://genome.ucsc.edu/).
(XLS)

File S1 **A.BED file corresponding to the genomic positions of the markers enables to export all genes possibly included in the amplified regions, according to UCSC genome draft, hg19 (** http://genome.ucsc.edu/ **).**
(BED)
